# Neural Correlates of Speech Segregation Based on Formant Frequencies of Adjacent Vowels

**DOI:** 10.1038/srep40790

**Published:** 2017-01-19

**Authors:** Claude Alain, Jessica S. Arsenault, Linda Garami, Gavin M. Bidelman, Joel S. Snyder

**Affiliations:** 1 Rotman Research Institute, Toronto, Ontario, Canada; 2 University of Toronto, Toronto, Ontario, Canada; 3 University of Memphis, Memphis, Tennessee, United States; 4 Department of Psychology, University of Nevada, Las Vegas, United States

## Abstract

The neural substrates by which speech sounds are perceptually segregated into distinct streams are poorly understood. Here, we recorded high-density scalp event-related potentials (ERPs) while participants were presented with a cyclic pattern of three vowel sounds (/ee/-/ae/-/ee/). Each trial consisted of an adaptation sequence, which could have either a small, intermediate, or large difference in first formant (Δ*f*_1_) as well as a test sequence, in which Δ*f*_1_ was always intermediate. For the adaptation sequence, participants tended to hear two streams (“streaming”) when Δ*f*_1_ was intermediate or large compared to when it was small. For the test sequence, in which Δ*f*_1_ was always intermediate, the pattern was usually reversed, with participants hearing a single stream with increasing Δ*f*_1_ in the adaptation sequences. During the adaptation sequence, Δ*f*_1_-related brain activity was found between 100–250 ms after the /ae/ vowel over fronto-central and left temporal areas, consistent with generation in auditory cortex. For the test sequence, prior stimulus modulated ERP amplitude between 20–150 ms over left fronto-central scalp region. Our results demonstrate that the proximity of formants between adjacent vowels is an important factor in the perceptual organization of speech, and reveal a widely distributed neural network supporting perceptual grouping of speech sounds.

Speech comprehension in noisy environments is constrained by our capacity to group sound elements coming from one source (i.e., one talker) and segregate these from other sources (i.e., other talker(s)). This “auditory scene analysis” is one of the most complex communication challenges that we engage in regularly. It allows us to transform the incoming acoustic waveform into “probable” auditory objects (i.e., mental representations of sounds) that correspond to the events in the external environment^1^,^2^,^3^,^4^. Although our perceptual system is generally successful at grouping speech sounds, even in noisy environments our understanding of how speech sounds are perceptually organized over time remains limited. Theories and models derived from studies using pure tone stimuli may not readily apply to speech, which is a naturally occurring and highly familiar stimulus. Exposure to a wide range of speech stimuli as well as many exemplars of the same speech token provides an ideal situation to learn about speech-specific properties (e.g., formant cues) and stimulus invariance that may assist in the successful perceptual grouping of speech stimuli. Spectro-temporally rich sounds, such as those used in spoken communication (e.g., vowels) often involve smooth fundamental frequency (ƒ_0_) and formant changes between adjacent phonemes that may play an important role in the perceptual organization of speech sounds. However, despite their high ecological validity, few studies have used well-controlled speech stimuli to induce stream segregation.

Dorman *et al*.^5^ were among the first to examine the influence of formant differences on streaming using repeating four-item vowel sequences. In their study, the vowels shared the same *f*_0_, but the order of the four vowels was manipulated to promote grouping based on the first formant (*f*_1_) differences between adjacent speech tokens. They found that the ability to perceive the items in the correct order was greater when smooth formant differences between the vowels were preserved. Misjudgment of repeating vowels was explained in terms of stream segregation, triggered by the discontinuity in formant transition of adjacent vowels^5^. Subsequent studies using three-6 or six-item^7^,^8^ vowel sequences have also shown that increasing the *f*_0_ difference of adjacent vowels promotes the segregation of sequences of vowels into two separate streams.

Animal studies and neuroimaging research in humans suggest that auditory stream segregation involves a widely distributed neural network that comprises brainstem, midbrain, primary and secondary auditory cortices as well as the inferior parietal lobule (IPL)^9^,^10^,^11^,^12^,^13^,^14^,^15^,^16^,^17^. Prior studies aiming to characterize the neural architecture supporting auditory stream segregation have used relatively simple sounds (e.g., pure tones) that are presented in an “ABA—ABA—” pattern in which “A” and “B” are tones of different frequencies and “—” is a silent interval. The greater the stimulation rate and frequency separation, the faster listeners are able to report hearing separate streams of sounds. The perception of two streams emerges progressively after the onset of the sequence and often fluctuates back and forth between one and two streams similar to bi-stable perception in vision^18^,^19^. Similarly, as the frequency separation increases between the A and B tones, the amplitude of the responses generated by the B tone also increases. Neurophysiological recordings in non-human primates^20^,^21^,^22^ and functional magnetic resonance imaging (fMRI) in humans^13^ provided converging evidence for increased activation in Heschl’s gyrus with increasing frequency separation. Scalp recordings of event-related potentials (ERPs) revealed an increase in sensory evoked response as a function of frequency separation, which occurs at about 100–300 ms after sound onset over the frontocentral scalp region and right temporal areas^23^. These ERP modulations appear to index a relatively automatic process as it is also present when participants are not actively paying attention to the stimuli^23^.

Notably, enhanced activity in auditory cortex can also be observed when perceiving two streams, as shown when magneto-encephalography (MEG) data were re-averaged as a function of the participants’ subjective perceptual experience (hearing “one” vs. “two” streams)^24^. Studies using fMRI have observed stimulus-driven effects^25^ as well as perceptual-related changes in IPL activity^12^, when participants reported hearing one versus two streams. Together these studies suggest that the perception of concurrent sound streams is associated with activity in auditory cortices and inferior parietal cortex. While the perceptual organization of speech sounds likely involves brain areas similar to those described for pure tones, one may also posit that the perceptual grouping of speech would engage more left-lateralized brain areas than those typically involved in grouping pure tone stimuli.

The present study aimed to identify neural correlates associated with the perceptual organization of speech sounds. We used a variant of the ABA- pattern in which pure tones were substituted with two different vowels (/ee/-/ae/-/ee/) that differed in *f*_1_ frequency while keeping the *f*_0_ (i.e., voice pitch) constant between vowels (Fig. 1). Vowel sequences provide a reliable and useful tool for investigating the perceptual organization of speech sounds that may otherwise be obscured by additional syntactic and semantic information present in sentences^26^. We adopted a paradigm from Snyder *et al*.^27^ in which participants are first presented with a short sequence of ABA pattern (i.e., adaptation sequence) that may have either small, intermediate, or large difference in *f*_1_ (Δ*f*_1_) frequency (Fig. 2). After a brief delay, participants are presented with another ABA pattern (i.e., test sequence) in which Δ*f*_1_ is always intermediate and usually yields an ambiguous percept^28^. Participants indicated whether they heard one or two streams after both the adaptation and test sequences. Prior research using pure tones has shown a greater likelihood to report hearing two streams after the adaptation with increasing Δ*f*_1_. At test, the effects of Δ*f*_1_ was reversed, with participants more likely to report hearing two streams when the test sequence was preceded by an ABA pattern with a small frequency separation^29^,^30^. Notably, ERPs elicited during the test sequence were modulated by both the physical manipulation and the perception of the adaption sequence^27^. Hence, using such a design allows us to examine changes in neural activity associated with Δ*f*_1_ as well as activity related to perception. We hypothesized that Δ*f*_1_ would be reflected in neural activity in auditory cortices. Prior neuroimaging research suggests a left hemisphere bias in processing the fine temporal structure of auditory stimuli^31^. Accordingly, we anticipated greater ERP modulations in left than the right hemisphere because the perceptual organization of speech sounds based on *f*_1_ differences depends on processing the fine temporal structure of the speech sounds. As in Snyder *et al*.^27^, we predicted that neural correlates reflecting the processing of Δ*f*_1_ frequency between adjacent vowels would differ from those related to the perception of concurrent streams of speech sounds.

## Results

### Behavioral data

Figure 3 shows the group-average proportion of the responses in which participants (N = 16) indicated hearing two auditory streams as a function of Δ*f*_1_ during the adaptation and test sequences. The effect of Δ*f*_1_ on perception following the adaptation and test sequences was assessed using a repeated-measures analysis of variance (ANOVA) with Δ*f*_1_ as the within-subject factor. For all ANOVAs reported, results of the pairwise comparisons were corrected for multiple comparisons using Bonferroni-adjusted contrasts (IBM SPSS Statistics 24).

For the adaptation sequence, the proportion of trials in which participants reported hearing two concurrent auditory streams increased with increasing Δ*f*_1_ (*F*(2,30) = 78.423, *p* < 0.001, η_*p*_^2^ = 0.839, all pair-wise comparison *p* < 0.001; linear trend: *F*(1,15) = 360.173, *p* < 0.001, η_*p*_^2^ = 0.960). For the test sequence, in which Δ*f*_1_ was always intermediate, the pattern was reversed. That is, there was a difference in perception at test based on which Δ*f*_1_ was presented at adaptation; participants were significantly *less* likely to report hearing two streams with increasing Δ*f*_1_ in the adaptation sequences (*F*(2,30) = 20.362, *p* < 0.001, η_*p*_^2^ = 0.576, all pair-wise comparison *p* < 0.05; linear trend: *F*(1,15) = 26.685, *p* < 0.001, η_*p*_^2^ = 0.640). These results demonstrate an effect of prior stimulus on perceptual organization of speech sounds analogous to previous behavioural findings observed with tonal stimuli^27^,^29^.

In order to investigate the impact of prior perception on subsequent classification, we compared the proportion of trials where participants reported streaming at test based on the perception of intermediate adaptation sequences. Figure 4 shows the proportion of trials where participants indicated hearing two streams at test when the ambiguous (i.e., intermediate Δ*f*_1_) adaptation sequence was heard as either one stream or two streams. For comparison, we show participants’ perception at test as a function of their perception for small and large Δ*f*_1_. This analysis shows that when Δ*f*_1_ does not change between adaptation and test sequence, participants are more inclined to report same percept as in the adaptation sequence, *t*(15) = 4.97, *p* < 0.001. This is markedly different from what was observed when the adaptation and test sequences had different Δ*f*_1_. That is, participants were more likely to report hearing two streams at test if they heard one stream during adaptation and vice versa, they more often indicated hearing one stream at test if they heard two streams during adaptation. In other words, participants’ perceptual decisions at test tended to switch when adaptation sequences had small or large Δ*f*_1_, but when prior Δ*f*_1_ was intermediate the participants were more inclined to report the same percept as in the preceding (i.e., adaptation) sequence.

### Electrophysiological data

Both adaptation and test sequence onset generated transient ERPs that comprised a positive (P1), a negative (N1) and a positive (P2) wave peaking respectively at about 60, 120, and 200 ms that were maximal at frontocentral scalp regions. These transient ERPs were followed by periodic fluctuations in ERP amplitude that corresponded closely with rate of stimulus presentation (steady-state responses). The effect of Δ*f*_1_ on neuroelectric activity was examined on epochs time-locked on the triplet onset. These segments of auditory steady-state responses showed a difference in amplitude at onset between the small and large Δ*f*_1_ conditions, which likely reflect remaining Δ*f*_1_-related activity from the previous triplet.

### Adaptation sequence

The electrophysiological data from one participant was excluded due to excessive artifacts during recording. We used a cluster analysis procedure and permutation-based statistics to test for the effect of Δ*f*_1_ on ERP amplitude (BESA Statistics 2.0). The analysis identified three significant clusters (Fig. 5). During the adaptation sequence, increased Δ*f*_1_ resulted in three ERP modulations between 245 and 500 ms after the onset of the first vowel (i.e., 95–350 after the second vowel onset). The latency of these modulations are comparable to that observed in a prior study using pure tone stimuli^23^,^24^. The first and second clusters revealed a left lateralized modulation over the temporal-parietal and temporal scalp regions, respectively, which may reflect activity from generators located in the superior temporal gyrus. Over the right frontal region, the small and intermediate Δ*f*_1_ generated ERPs with comparable amplitude. The third cluster corresponded to an ERP modulation that peaked at about 250 ms after the onset of the second vowel, with a more gradual change in ERP amplitude as a function of Δ*f*_1_. This ERP modulation showed a polarity reversal between the fronto-central scalp region and posterior inferior parietal and occipital areas, which is consistent with generators in auditory cortices along the Sylvian fissure.

We used Classical LORETA (Low Resolution Electromagnetic Tomography) Analysis Recursively Applied (CLARA, BESA version 6.1) to estimate source activity associated with processing Δ*f*_1_. This distributed source modeling approach estimates the total variance of the scalp-recorded data. It uses a smoothness constraint, which ensure that current changes little between adjacent regions in the brain^32^,^33^. In the present study, the voxel size in Talairach space was 7 mm and the regularization parameters, which account for the noise in the data, was set at 0.01% singular value decomposition. The source analysis was performed at each time point from the difference wave between ERPs elicited by small or large Δ*f*_1_. The source solution was inspected visually for several time points and was considered stable if the dominant source remained constant over a 30 ms interval (i.e., 15 ms before and after the peak). The results are displayed on a standard MRI from BESA (version 6.1). We identified three primary sources of activity, one for each cluster. The processing of Δ*f*_1_ in the first cluster was associated with source activity in the middle and anterior portions of the right temporal lobe. The second cluster was associated with activity that was strongest in the left temporal region near Heschl’s gyrus. For the third cluster, source activity near the right temporal-parietal junction peaked at about 450 ms after the triple onset.

### Test sequence

Figure 6 shows the effects of adaptation ∆*f*_1_ on ERPs elicited during test. The analysis revealed two significant clusters. When the adaptation sequence comprised a small ∆*f*_1_ (i.e., 1 stream), the ERPs at test showed a significant increase in positivity between 170–300 ms over left fronto-central scalp region after the first vowel of the triplet (i.e., 20–150 ms after the /ae/ vowel). This ERP modulation showed a polarity reversal between fronto-central scalp regions and mastoid electrodes. The distributed source analysis suggests contribution of generators located in the left prefrontal cortex as well as anterior portion of middle temporal gyrus bilaterally.

We also examined the impact of prior perception on ERPs by averaging responses during test as a function of the prior perception for only the ambiguous (intermediate Δ*f*_1_) sequences during adaptation. Data from two participants were excluded because of insufficient trials in one of the conditions (perception of one vs. two streams). The effect of prior perception on ERP amplitude during the test sequence was not significant. Lastly, we compared ERPs as a function of perception at test regardless of the prior sequence. This analysis revealed an early modulation between 10 and 80 ms after triplet onset at fronto-central scalp sites (*p* < 0.001) when participants reported hearing two streams as opposed to one stream (see Supplementary Material).

### Correlations

Bivariate correlations between mean audiometric thresholds from 250 to 8000 Hz pure tone thresholds, QuickSIN scores (i.e., speech-in-noise perception), and the probability of hearing two streams (“streaming”) were examined to explore the relationship between the perceptual organization of speech sounds, hearing sensitivity, and degraded speech comprehension skills. The correlation between mean audiometric threshold and streaming was not significant (*r* = −0.024, *p* = 0.929), nor was the correlation between QuickSIN and the subjective measure of streaming (*r* = −0.438, *p* = 0.090). The correlation between mean audiometric thresholds and QuickSIN was not significant (*r* = −0.367, *p* = 0.162). These results are expected given the young, normal hearing demographics of our cohort.

We also examined the relation between ERP amplitude and perception. For each participant, a correlation coefficient was calculated between the changes in perception as a function of Δ*f*_1_ and ERP mean amplitude (Fig. 7). The significance of these correlations was then examined through a *t* test on the group mean correlation. For each cluster, the mean amplitude measurements (50 ms centered on the peak latency) included all electrodes from the cluster (see Fig. 5). For Cluster 1, a significant positive correlation was found between ERP amplitude and perceptual judgment (*r* = 0.68, *t*(14) = 7.06, *p* < 0.001). For Cluster 2, the correlation was negative (*r* = −0.64, *t*(14) = 4.12, *p* = 0.001). For Cluster 3, the correlation between ERP amplitude and perception was positive (*r* = 0.60, *t*(14) = 4.22, *p* = 0.001). We also observed a significant correlation between ERP amplitude from Cluster 1 and 2 (*r* = −0.54, *t*(14) = 3.31, *p* = 0.005). The group mean correlation between Cluster 1 and 3 was not significant (*r* = 0.35, *t*(14) = 1.91, *p* = 0.08), nor was the group mean correlation between Cluster 2 and 3 (*r* = −0.33, *t*(14) = 1.75, *p* = 0.10). These individual correlations suggest a link between ERP measures and perceptual organization of speech sounds.

Lastly, for each cluster we examined whether the participants’ mean amplitude (using the difference in ERP amplitude between small and large Δ*f*_1_) correlated with Quick SIN score or pure tone thresholds. None of these correlations were significant.

## Discussion

In the present study, perceptual grouping of speech sounds was promoted by increasing the first-formant frequency separation between adjacent vowels. The *f*_1_ difference between successive vowels was relatively small in comparison to the more typical frequency differences used in pure tone, ‘ABA’-like sequences^23^,^24^,^27^. Yet, these relatively small Δƒ_1_ frequency changes yield significant differences in perceptual organization. This highlights the significance of Δƒ_1_ in perceptually organizing speech sounds. Our results are in agreement with previous studies using speech sounds^5^,^6^,^7^,^8^,^28^,^34^, which have shown that participants are more inclined to report hearing two concurrent streams when formant differences between consecutive vowels are large or intermediate than when they are small. An abrupt change in formant frequency may promote the separation of phonetic segments and increase the perceptual segregation of speech tokens into two separate auditory streams. Participants may have also used rhythmic cues to guide their decisions about streaming, with the perceived galloping rhythm typical of ABA- paradigms decreasing with increasing first formant separation between adjacent stimuli.

The effects of formant proximity on speech segregation were associated with changes in ERP amplitude that were consistent with activity arising from the primary and associative auditory cortices along the Sylvian fissure. During the adaptation sequence, the first modulation peaked at about 150 ms after the onset of the second vowel within an ABA- paradigm (i.e., ~275 ms from triplet onset, Fig. 5). The latency of this modulation was comparable to that of prior studies using pure tones^23^,^27^, and may reflect a modulation of the P2 wave. The P2 wave has been associated with speech discrimination^35^,^36^, and may index categorical speech perception^37^ and sound object identification^38^,^39^. We also found a second left-lateralized modulation that peaked at about 175 ms after the onset of the second vowel within the ABA- triplet (i.e., ~325 after triplet onset) as well as another modulation peaking at 250 ms (i.e., ~445 ms after triplet onset) over the fronto-central scalp region. These two modulations have not been previously described in prior studies using pure tone stimuli^23^,^27^, and may be specific to speech processing. A similar “post-P2” wave was observed in a speech categorization task^37^, which varied with perceptual (rather) than acoustic classification and could represent integration or reconciling the input with a memory template. During adaptation, there were some differences in ERP amplitude at the onset of the triplet between small and large Δƒ_1_ conditions, which may reflect activity from the previous repetition within the steady-state sequence. In the present study, difference in neural activity prior to or immediately after triplet onset was heightened by the baseline correction, which was chosen to highlight transient activity time-locked on the /ae/ vowel.

In the present study, the left-lateralized response may index processing of acoustic details of the first formant whereas the latter modulation could reflect post-perceptual categorization processes or streaming-related activity. The mid temporal and temporal-parietal junction are part of the ventral and dorsal stream processing of speech^40^,^41^,^42^, and may provide acoustic representations in sensorimotor interface areas located in the left posterior STG and/or IPL to constrain perception. Importantly, the neural network supporting speech segregation appears to differ substantially from that observed for pure tone stimuli, with more pronounced activity in the left hemisphere and additional processing associated with the segregation of adjacent vowels into two separate streams. Based on behavioural evidence, Remez *et al*.^43^ argued that perceptual organization of speech sounds must involve a specific pathway because it seems to escape primitive perceptual organization rules. Although our ERP findings provide some support for distinct pathways supporting the perceptual organization of speech sounds, further research is needed to extend this novel finding to more complex listening situations involving words and sentences.

Interestingly, prior stimulus presentation that yielded a clear percept seemed to bias perception of an incoming ambiguous stimulus *away* from what was just heard (contrastive context effect), while prior perception of ambiguous stimuli seemed to prime perception *towards* the perceived perceptual organization of the stimuli (facilitative context effect). Our findings are analogous to those of prior research using tonal stimuli^27^,^29^,^30^,^44^. Although different neural mechanisms may underlie stimulus-related (i.e., Δ*f*_1_) and perception-related (i.e., one stream vs. two streams) context effects, both context effects recede over time at a similar rate^44^. Further research is required to determine whether similar mechanisms are responsible for speech sound segregation as in tonal segregation, as well as whether the streaming of speech sounds is affected by factors such as attention and prior knowledge^10^.

One of the fundamental processes of the human auditory system is to organize sounds into meaningful elements, such as separating a police siren from the music playing through a car radio, or identifying and attending to a friend’s voice in a noisy room. The findings of the current study support the notion that auditory stream segregation of speech sounds is impacted by context. We also found a small ERP modulation as a function of perception at test. That is, listeners’ perception of one and two streams at test was associated with a distinct neural signature. This ERP modulation peaked at about 60 ms after triplet onset and preceded the one observed in a prior study using pure tones by about 60 ms^27^. However, we should be cautious while interpreting this (unexpected) finding, which was significant only when data from the small and large Δ*f*_1_ condition were included in the analysis. Further research is needed to replicate these small changes in neural activity associated with the perception of concurrent streams of speech sounds. It will also be important in future studies to examine whether these changes vary as a function of the cue used to promote the segregation of speech sounds. Using vowels presented simultaneously, Du *et al*.^45^ observed different patterns of activity when the vowels were segregated based on difference in fundamental frequency and location, consistent with the dual pathways model of audition^41^.

In the current study, we did not find an association between performance during the speech-in-noise test and subjective measures of stream segregation nor ERP amplitude. This result differs from those reported by Mackersie, Prida, and Stiles^46^, who found a significant correspondence between streaming judgment and simultaneous sentence perception. It is notable that Mackersie *et al*.^46^ used a broader age range (young and older adults), as well as a broader range of hearing ability as measured with pure-tone thresholds. In other words, their sample was more heterogeneous than the one used in the present study. Indeed, our sample of young adults was fairly homogenous with respect to hearing ability and age, thereby reducing the variability in responses and our capacity to observe non-zero correlations between tasks. Our findings also differ from those of Gaudrain *et al*.^47^, who observed a significant correlation between listeners’ performance in an order-naming task on vowel sequence and their ability to identify monosyllabic words embedded in time-reversed speech from a single talker. There are several factors that could account for this discrepancy. These include the method used to infer streaming and the task used to assess speech-in-noise reception and comprehension. In the present study, the lack of relationship between QuickSIN scores and subjective streaming responses suggests that different perceptual and/or cognitive processes were engaged during the experimental tasks and the speech-in-noise test. For example, the QuickSIN may rely more on cognitive (rather than perceptual) processes, such as attention, working memory and linguistic processing, while the subjective measures used in the present study are more perceptual-based. Future studies could incorporate measures of attention and working memory to explore this relationship further.

In summary, using complex, ecologically valid stimuli, we have shown that speech sounds can be grouped based on first formant differences between adjacent speech sounds. Importantly, the analysis of EEG data reveals transient changes in neural activity that are sensitive to first formant difference as well as perceptual context. This study adds to the rich volume of literature characterizing the phenomenon of streaming and provides a new neural metric to assess perceptual organization of speech sound in healthy individuals as well as those who may experience problems in understanding speech in multi-talker environment.

## Material and Methods

### Participants

Eighteen healthy young adults were recruited from Baycrest Health Sciences participant database. Two were excluded due to technical problems during data acquisition. The final sample included 16 participants (M_age_ = 23.25 yr, SD = 4.39; 8 females). All participants were right-handed except for one who was left-handed. All were fluent English-speakers with no known neurological or psychiatric issues and no history of hearing or speech disorders. The study was carried out in accordance with relevant guidelines and regulations and was approved by the University of Toronto and Baycrest Hospital Human Subject Review Committee. Participants gave informed written consent before taking part in the study and received a small honorarium for their participation.

### Stimuli and Task

**Figure 1:**
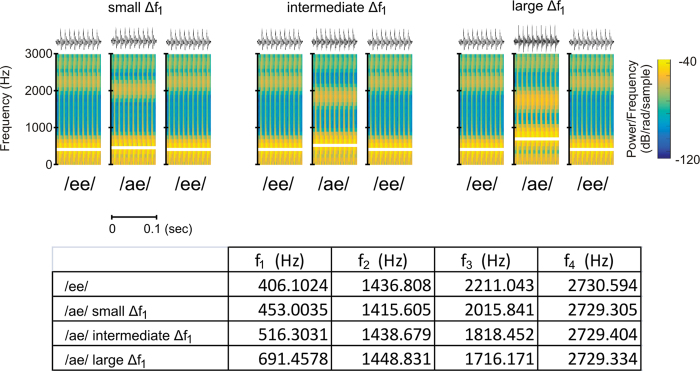
Top. Spectograms of the vowels used during small, intermediate and large difference in first formant frequency (Δƒ_1_). The white horizontal lines highlight first formant frequency within the spectrogram. Bottom. Table showing the actual frequency of the first, second, third and fourth formants for the vowel /ee/ and /ae/ for small, intermediate and larger Δƒ_1_.

**Figure 3:**
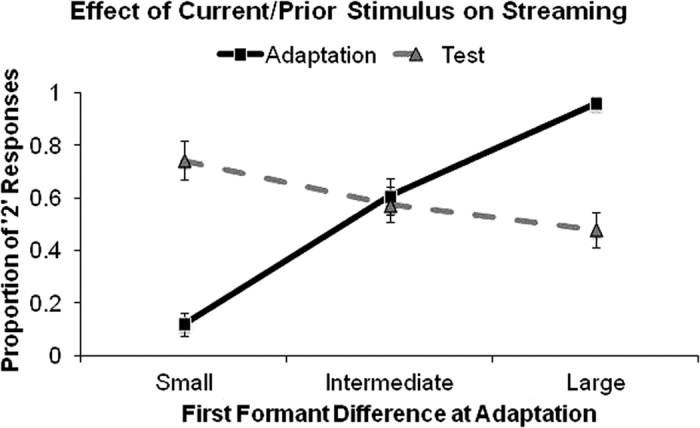
Effects of first formant differences on perception of streaming during the adaptation and test phase. Note that the difference in first formant frequency between adjacent vowels presented at test is always intermediate. Error bars represent standard error of the mean.

**Figure 4:**
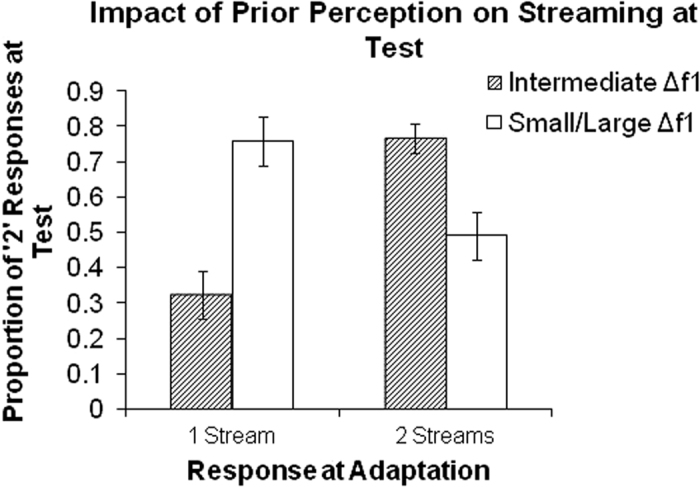
Effect of perception during adaptation on streaming reports at test for ambiguous (intermediate ∆*f*_1_) and non-ambiguous (small/large ∆*f*_1_) adaptation sequences. For comparison, we show the proportion of trials perceived as two streams depending on perception after the adaptation. Error bars represent standard error of the mean.

**Figure 5:**
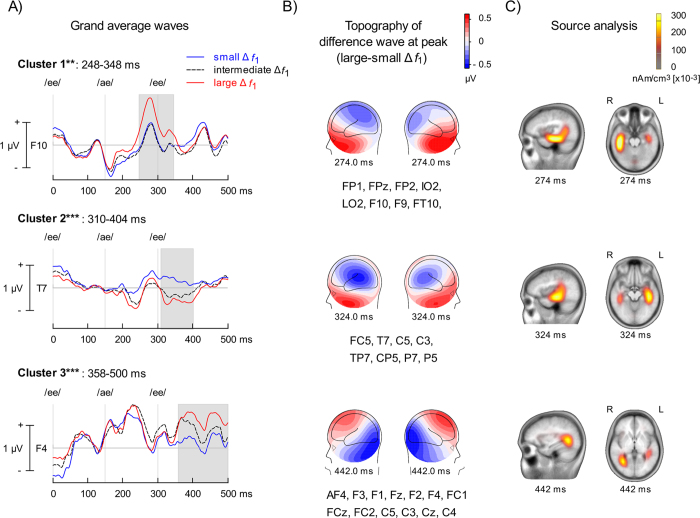
Adaptation phase. (**A**) Group mean event-related potentials (ERPs) time-locked on triplet onset when the difference between the first formant (Δ*f*_1_) was small (blue) or large (red). Vertical lines indicate the onsets of the corresponding vowel in the triplet. Note that baseline correction was applied prior to the /ae/ vowel rather than triplet onset to emphasize transient changes in neural activity following the changes in Δ*f*_1_. Three ERP modulations (i.e., clusters) were identified. The third cluster shows difference at the triplet onset, which likely reflects residual Δ*f*_1_-related changes in ERP amplitude from the previous triplet. Each panel shows the recording site (i.e., electrode) where the difference was largest for each cluster. The shaded area reveals the time window that was significantly different within each cluster. (**B**) Left and right views of iso-contour maps showing the peak of the ERP modulation as revealed by the difference in ERPs elicited by small and large Δ*f*_1_. The electrodes showing significant effects of Δ*f*_1_ are listed below the contour maps. The blue color refers to negative voltage while the red color indicates positive voltage. (**C**) Cortical Low resolution electromagnetic tomography Analysis Recursively Applied (CLARA, BESA version 6.1) at each peak activity identified in the cluster analysis. **p < 0.01, ***p < 0.001.

**Figure 6:**
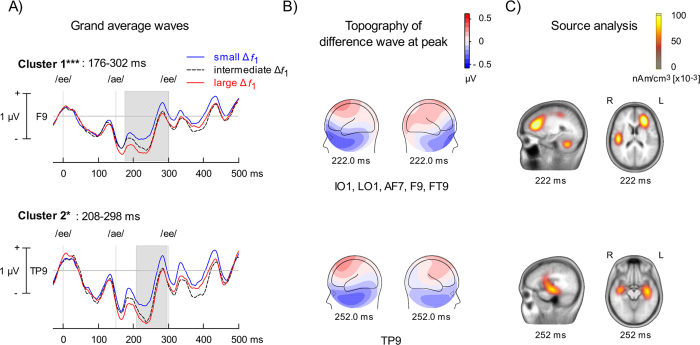
Test phase. (**A**) Group mean event-related potentials (ERPs) time-locked on triplet onset when the test sequence was preceded by small or large difference between the first formant (Δ*f*_1_). Vertical lines indicate the onsets of the corresponding vowel in the triplet. Note that baseline correction was applied prior to the triplet onset. Two ERP modulations (i.e., clusters) were identified. The top and bottom panels show the recording site (i.e., electrode) where the difference was largest for each cluster. The shaded area revealed the time window that was significantly different within each cluster. (**B**) Left and right views of iso-contour maps showing the peak of the ERP modulation as revealed by the difference in ERPs at test when preceded by small and large Δ*f*_1_. The electrodes showing significant effects of Δ*f*_1_ are listed below the contour maps. The blue color refers to negative voltage while the red color indicates positive voltage. (**C**) Cortical Low resolution electromagnetic tomography Analysis Recursively Applied (CLARA, BESA version 6.1) at each peak activity identified in the cluster analysis. *p < 0.05, ***p < 0.001.

**Figure 7:**
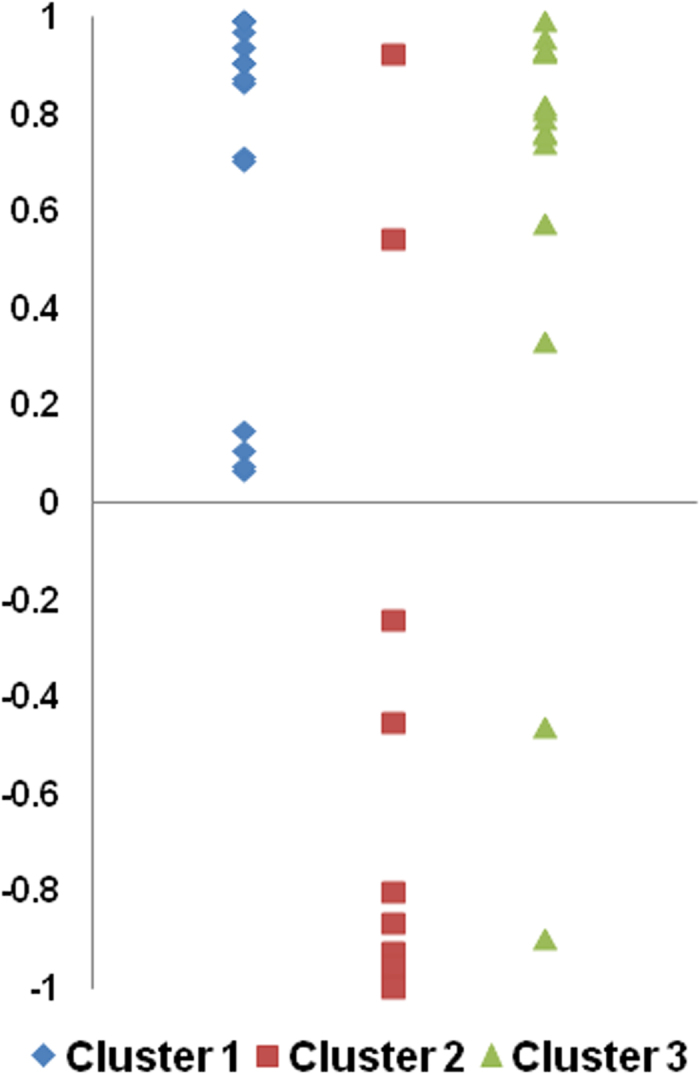
Scatterplots displaying the Pearson correlations (y axis) between participants’ perception of streaming and event-related potential (ERP) mean amplitude for the adaptation sequence. For each cluster, the mean amplitude measurements (50 ms centered on the peak latency) included all electrodes from the cluster (see Fig. 5).

Stimuli consisted of synthetic vowel sounds /i/ (as in see) and /ae/ (as in cat), henceforth referred to as “ee” and “ae” (Fig. 1). Vowel stimuli were synthesized using a cascade formant synthesizer implemented in MATLAB^48^ using a sampling rate of 48828 Hz. Each token contained an identical voice fundamental (*f*_0_ = 100 Hz). The first formant frequency difference (Δ*f*_1_) between /ee/ and /ae/ was either small (Δ*f*_1_ = 47 Hz), intermediate (Δ*f*_1_ = 110 Hz), or large (Δ*f*_1_ = 285 Hz), where *f*_1_ of /ee/ was fixed at 400 Hz and *f*_1_ of /ae/ was allowed to vary (Fig. 1). These values were chosen based on a prior study showing that they typically yield perception of one stream, ambiguous, or two streams, respectively^28^. They correspond to an 11.75, 27.50, and 71.25% increase in *f*_1_ frequency relative to its original value. Each vowel was 100 ms in duration, and were presented binaurally at 75 decibel (dB) sound pressure level through Sennheiser HD 265 headphones.

The vowels were presented in an ABA- pattern as/ee/-/ae/-/ee/, with first formant frequency differences between /ee/ (A) and /ae/ (B). Only the second vowel within the triplet was manipulated. The inter-stimulus interval (ISI) between /ee/ and /ae/ was always 50 ms whereas the ISI between triplets was fixed at 100 ms. Each trial consisted of an adaptation sequence, which could have either a small, intermediate, or large Δ*f*_1_, followed by a test sequence, in which Δ*f*_1_ was always intermediate. The adaptation acted as a priming stimulus to bias perception at test. Both sequences were seven seconds in duration, and were separated by 1.44 seconds of silence. In each phase, 14 repetitions of the /ee/-/ae/-/ee/- triplets were presented sequentially. After each sequence (adaptation or test), participants pressed one of two keys on a response box (Tucker-Davis Technologies) indicating whether the previous sequence was perceived as one or two streams.

Participants were seated in a comfortable chair in a sound-attenuated chamber for the duration of the study. The testing session began with two hearing tests – the pure tone thresholds audiometry (hearing thresholds) and the QuickSIN^49^ (speech-in-noise recognition). The order of the two tests was counter-balanced across participants. Participants were then prepared for EEG testing (see below) and the concept of streaming was explained to participants. A brief practice session was given in order to familiarize participants with the stimuli and task. Participants were encouraged to keep their eyes fixated in a comfortable position and listen to the sounds. Participants completed five blocks of 30 trials each for a total of 150 trials, with each Δ*f*_1_ condition (small, intermediate, large) being presented 50 times throughout the study. In each block of trials, the three levels of Δ*f*_1_ were presented in a random order. Each participant was presented with 1800 triplets at adaption (600 for each level of Δ*f*_1_) and at test (1800 for intermediate Δ*f*_1_).

### Recording of Neuroelectric Brain Activity

The electroencephalogram was digitized continuously (sampling rate 500 Hz) from an array of 64 electrodes with a bandpass filter of 0.05–100 Hz using NeuroScan Synamps2 (Compumedics, El Paso, TX, USA). Eye movements were monitored with electrodes placed at the outer canthi and at the inferior orbits. During recording, all electrodes were referenced to the vertex electrode (i.e., Cz). For off-line data analysis, they were re-referenced to an average reference. For each participant, a set of ocular movements was obtained prior to and after the experiment^50^. From this set, averaged eye movements were calculated for both lateral and vertical eye movements as well as for eye-blinks. A principal component analysis of these averaged recordings provided a set of components that best explained the eye movements. The scalp projections of these components were then corrected from the experimental ERPs in order to minimize ocular contamination, using Brain Electrical Source Analysis (BESA 6.0). Epochs contaminated by excessive deflections (greater than ± 120 uV anywhere in the epoch) after correcting for ocular contaminations were excluded from the averages. For each participant, the remaining epochs were averaged according to electrode position.

We created different averages for examining changes in neural activity associated with variation in stimulus ƒ_1_ acoustic and that associated with perception. Changes in neural activity associated with Δ*f*_1_ were examined by averaging /ee/-/ae/-/ee/- triplets from the adaptation sequence time-locked on the first vowel of the triplet. We have excluded the ERPs from the first triplet of the sequence because it generated a transient onset response that overlapped with the steady state responses. The last triplet of the sequence was also excluded from the analysis. The analysis epoch was defined as 0 to 500 ms after the onset of the first vowel from the /ee/-/ae/-/ee/- pattern. To facilitate the comparison with our prior study using tonal stimuli^27^, we used the same pre-stimulus baseline corrections. For assessing the impact of Δ*f*_1_ on neural activity, the ERPs were baselined using the 120–150 ms interval, which preceded the onset of the vowel that varied on ƒ_1_ (i.e., /ae/ vowel from the /ee/-/ae/-/ee/- pattern). Exploring the ERP correlates of perception we defined epochs as −30 to 500 ms during the test phase. We applied baseline correction for −30 to 0 ms to enable detecting responses corresponding to the pattern of the whole sequence. Changes in neural activity associated with perception were examined by averaging triplets from the test sequence, which had a constant ƒ_1_ separation throughout the experiment. The epochs were sorted based on the participants’ responses immediately after the test sequence, as well as prior responses given after the adaptation sequence.

All ERPs were digitally filtered to attenuate frequencies above 30 Hz (12 dB/octave; zero phase) prior to a cross subject statistical analysis of ERP amplitude using BESA Statistics 2.0. A two-stage analysis first computed a series of t-tests that compared the ERP amplitude between the conditions at every time point from 0 to 500 ms after triplet onset. This preliminary step identified clusters both in time (adjacent time points) and space (adjacent electrodes) where the ERPs differed between the conditions. In the second stage of this analysis, permutation tests were performed on these clusters. The permutation test used a bootstrapping technique to determine the probability values for differences between conditions in each cluster. The final probability value computed was based on the proportion of permutations that were significant for each cluster, and implicitly corrected for multiple comparisons. We used a cluster alpha of 0.05, one thousand permutations and clusters defined using a channel distance of 4 cm, which resulted in an average of 5.08 neighbors per channel.

## Additional Information

**How to cite this article:** Alain, C. *et al*. Neural Correlates of Speech Segregation Based on Formant Frequencies of Adjacent Vowels. *Sci. Rep.* **7**, 40790; doi: 10.1038/srep40790 (2017).

**Publisher's note:** Springer Nature remains neutral with regard to jurisdictional claims in published maps and institutional affiliations.

## Supplementary Material

Supplementary Information

## Figures and Tables

**Figure 2:**
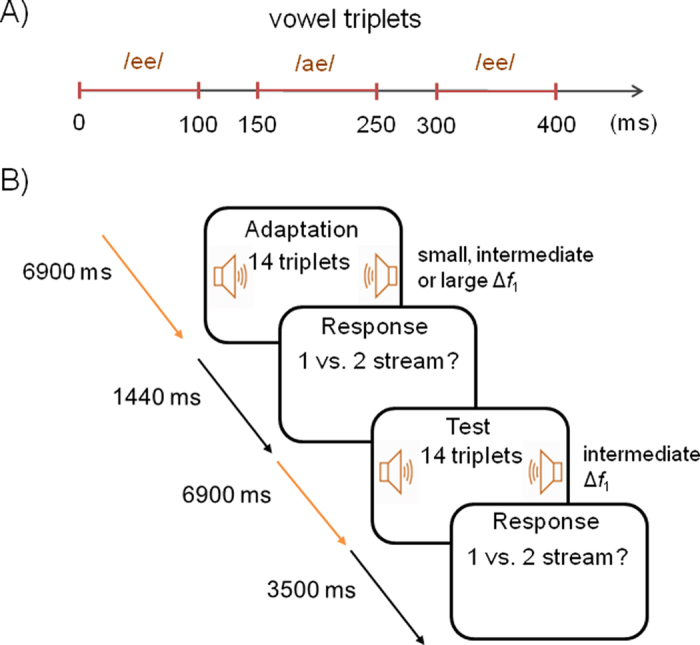
(**A**) Graphical depiction of vowel pattern. Each triplet lasted 400 ms and contained three vowels. The interval between triplets was 100 ms. When the first formant difference between consecutive vowels was small, the sequence was usually heard as a single galloping rhythm. (**B**) Schematic of a trial. Each trial consisted of an adaptation sequence of 14 triplets followed by a test sequence of 14 triplets, each requiring the participant to make a response immediately after the sequence indicating whether one stream or two streams were perceived.
