# Supplementary material for: Neural Correlates of Speech Segregation Based on Formant Frequencies of Adjacent Vowels
Source: Sci Rep. 2017 Jan 19;7:40790. doi: 10.1038/srep40790 (PMC5244401; doi:10.1038/srep40790)
Supplement: Supplementary Information [file srep40790-s1.pdf]

Supplementary material.

Neural correlates of speech segregation based on formant frequencies of adjacent vowels.

Claude Alain, Jessica S. Arsenault, Linda Garami, Gavin M. Bidelman, and Joel S. Snyder

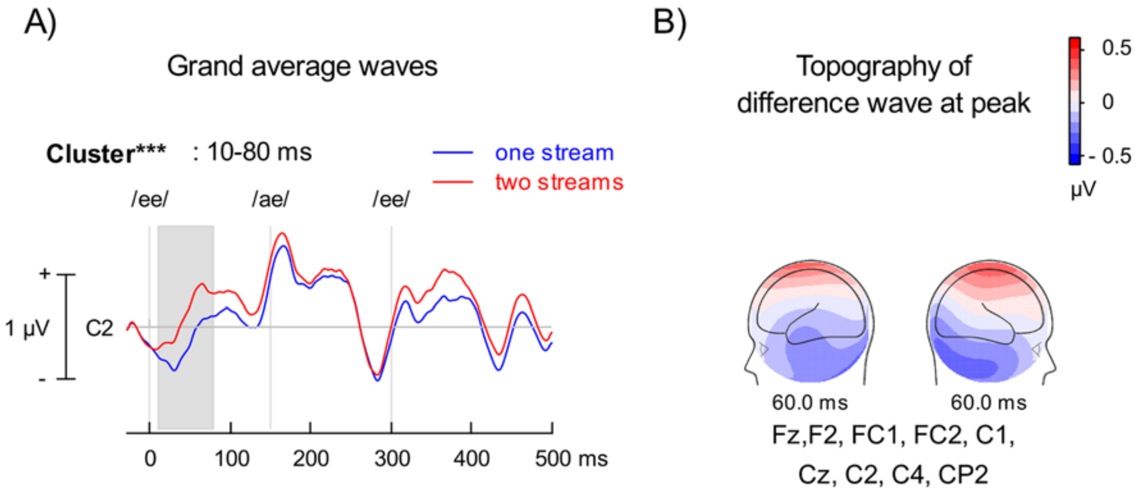

Test phase. A) Group mean event-related potentials (ERPs) time-locked on triplet onset when the adaptation sequence preceding the test sequence was perceived as one or two streams. The ERPs associated with one or two streams were created by averaging trials from the small, intermediate, and large Δf_1 conditions. Vertical lines indicate the onsets of the corresponding vowel in the triplet. Note that baseline correction was applied prior to the triplet onset. One ERP modulations (i.e., cluster) was identified. The shaded area revealed the time window that was significantly different between the one stream and two streams percept. B) Left and right views of iso-contour maps showing the peak of the ERP modulation as revealed by the difference in ERPs at test when the adaptation sequence was heard as one or two streams. The electrodes showing significant effects of perception are listed below the contour maps. The blue color refers to negative voltage while the red color indicates positive voltage.
